# A proposed definition of participatory organizational interventions

**DOI:** 10.1002/1348-9585.12386

**Published:** 2023-02-03

**Authors:** Asuka Sakuraya, Mako Iida, Kotaro Imamura, Emiko Ando, Hideaki Arima, Hiroki Asaoka, Hisashi Eguchi, Yui Hidaka, Ayako Hino, Akiomi Inoue, Reiko Inoue, Mai Iwanaga, Yuka Kobayashi, Yu Komase, Yasumasa Otsuka, Natsu Sasaki, Akihito Shimazu, Kanami Tsuno, Kazuhiro Watanabe, Norito Kawakami, Akizumi Tsutsumi

**Affiliations:** ^1^ Department of Digital Mental Health, Graduate School of Medicine The University of Tokyo Tokyo Japan; ^2^ Department of Psychiatric Nursing, Graduate School of Medicine The University of Tokyo Tokyo Japan; ^3^ Center for Cancer Control and Information Services National Cancer Center Tokyo Japan; ^4^ Shinagawa Ekimae Mental Clinic Tokyo Japan; ^5^ Department of Mental Health Institute of Industrial Ecological Sciences, University of Occupational and Environmental Health, Japan Kitakyushu Japan; ^6^ Department of Mental Health, Graduate School of Medicine The University of Tokyo Tokyo Japan; ^7^ Japan Society for the Promotion of Science Tokyo Japan; ^8^ Institutional Research Center, University of Occupational and Environmental Health, Japan Kitakyushu Japan; ^9^ Department of Public Health Kitasato University School of Medicine Sagamihara Japan; ^10^ Department of Community Mental Health and Law National Institute of Mental Health, National Center of Neurology and Psychiatry Tokyo Japan; ^11^ Department of Clinical Psychology, Faculty of Social Policy & Administration Hosei University Tokyo Japan; ^12^ Faculty of Human Sciences University of Tsukuba Tokyo Japan; ^13^ Faculty of Policy Management Keio University Fujisawa Japan; ^14^ School of Health Innovation Kanagawa University of Human Services Kawasaki Japan

**Keywords:** employee participation, occupational health, participatory organizational interventions

## Abstract

Participatory organizational interventions offer an effective way to promote occupational safety and health. Despite an increasing number of studies, a common definition of participatory organizational interventions has yet to be established. Therefore, we aimed to form a definition using the following process. First, we developed a tentative draft definition of organizational interventions and participatory elements, based on the relevant literature. The tentative definition was revised in several rounds of an extensive discussion by the authors. This resulted in the draft definition. We asked 15 selected international experts in occupational safety and health to review and comment on the draft definition. We carefully reviewed their comments, and formulated our final proposed definition. To summarize the key points of the final version of the definition, organizational interventions are planned actions that primarily directly target working conditions with the aim of promoting and maintaining of the highest degree of physical, mental, and social well‐being of workers in all occupations. In addition, as participatory elements of organizational interventions in the final definition, ideally, all workers participate in every step of the intervention, while participating in part of the steps of the intervention in some cases. Furthermore, in principle, all workers participate in each step of intervention, while it is also acceptable that only elected representatives among workers participate in the intervention.

## 1 INTRODUCTION

According to the International Labour Organization (ILO) and World Health Organization (WHO), occupational health is defined as “the promotion and maintenance of the highest degree of physical, mental, and social well‐being of workers in all occupations by preventing departures from health, controlling risks and the adaptation of work to people, and the people to their jobs”.^1^ Its main objectives are as follows: “(i) the maintenance and promotion of workers' health and working capacity; (ii) the improvement of working environment and work to become conducive to safety and health, and (iii) development of work organizations and working cultures in a direction which supports health and safety at work and in doing so also promotes a positive social climate and smooth operation and may enhance productivity of the undertakings.”^2^

To achieve the goal of occupational safety and health activity, a safe and healthy working environment should be ensured for all workers, as provided in ILO Conventions No. 155 and No. 161.^3^, ^4^, ^5^ In this framework, occupational safety and health, a discipline dealing with the prevention of work‐related injuries and diseases as well as the protection and promotion of the health of workers, aims at the improvement of working conditions and environment.^3^, ^6^ Thus, organizational interventions should be encouraged for promoting occupational safety and health.

Organizational interventions are group‐, team‐, or workplace‐based approaches to improve working conditions and environment.^7^, ^8^, ^9^, ^10^, ^11^ In conducting organizational interventions, the participation of workers has been strongly recommended by many researchers.^9^, ^11^, ^12^, ^13^, ^14^ For example, the recently published International Organization for Standardization (ISO) 45003 guideline states that the participation of workers is an essential component for the development, planning, implementation, maintenance, evaluation, and continual improvement of healthy and safe workplaces as well as the success of the process to manage psychosocial risk.^13^ The participation of workers is important because it increases worker control, sense of fairness, justice, and support; in addition, it helps to optimize the fit of the intervention to the organizational culture and context.^9^ However, participatory organizational interventions are characterized or defined in a various way.^9^, ^11^, ^12^, ^14^ For example, Nielsen et al. (2010) and Abildgaard et al. (2018) suggested that definitions of this type of intervention fail to agree on who participates in the intervention (e.g., all the workers or elected representatives), or the steps of the intervention in which workers participate.^9^, ^12^

Despite an increasing number of participatory organizational intervention studies,^15^, ^16^, ^17^ a comprehensive picture of this type of intervention and evidence for its overall effectiveness have not been well integrated. This is partly because there have been a limited number of systematic reviews and meta‐analyses specifically targeting this type of intervention. Lack of a common definition of participatory organizational interventions may make it difficult to conduct such an integration effort.

In this opinion paper, we aimed to propose a definition of participatory organizational interventions. Here, we formulated participatory organizational interventions as organizational interventions that include participatory elements. Having a commonly accepted definition would enable researchers and practitioners to conduct a variety of participatory organizational interventions under the same range of definitions and more easily integrate evidence of effectiveness or other program characteristics of participatory organizational interventions to develop a standard guideline for this type of approach.

## 2 METHODS

First, we developed a tentative draft definition of organizational interventions and participatory elements, based on relevant literature.^7^, ^8^, ^9^, ^10^, ^11^, ^12^, ^13^, ^14^, ^18^ Because there was not a common definition, we referred to six reviews related to participatory organizational intervention^7^, ^8^, ^9^, ^10^, ^11^, ^12^ and three important guidelines of occupational safety and health^13^, ^14^, ^18^ The tentative definition was revised in several rounds of an extensive discussion by the authors. This resulted in the draft definition. We asked selected international experts in this field to review and comment on the draft definition. We carefully studied their comments, and formulated our final proposed definition.

More specifically, from our social network, we invited 15 experts in this field who were active in research of organizational interventions or were involved in activities of international organizations related to occupational safety and health, such as ILO. These included academic researchers [e.g., professor or associate professor of university, and researchers at research institutes (*n* = 11), international organization staff (*n* = 3), and an occupational health consultant (*n* = 1)]. We sent the descriptions of the draft definition listed in the Results section to them and asked them to review and comment back to us. In the final step, we studied and integrated comments from the experts and formulated a comprehensive definition of participatory organizational interventions.

## 3 RESULTS

### 3.1 The draft definitions

1. Organizational interventions

We developed a draft definition of organizational interventions as follows. This was sent for review by 15 international experts,

*Organizational interventions are planned actions that directly target working conditions with the aim of preventing deterioration in mental health, physical health, quality of life, and work‐related outcomes of workers, and of promoting these outcomes. Organizational interventions are often primary and secondary prevention‐focused, but may also include tertiary prevention, for example, interventions to help return‐to‐work of workers with mental health problems*.

*Note*

- *Interventional actions may target single or multiple known risk factors at work (*e.g., *job content, workload and work pace, work schedule, job control, work environment and equipment, organizational culture and function, interpersonal relationships at work, role in organization, career development, home‐work interface)*.
- *Interventions, approaches applied to teams*.
- *Interventions may be multimodal, including any combination of individual, manager, and organizational approaches such as workplace mental health promotion with an organizational component*.
- *NOT: National, regional or global‐level policies, regulations; interventions which only address individual‐level interventions, or which only address knowledge, awareness, attitudes, stigma reduction of managers, or which only address knowledge, awareness, attitudes, stigma reduction of workers*.

2. Participatory elements of organizational interventions

To become a participatory organizational intervention, an organizational intervention is required to have the following participatory elements of interventions. This was also sent to the experts.

1. *Employees should participate in each step of intervention: they should be involved in action planning, implementing, evaluating, and reviewing the intervention itself*.
2. *In principle, all the employees in the workplace should participate directly in each step of intervention. However, indirect participation through elected representatives among employees could also be acceptable*.

### 3.2 Comments from experts and our accommodations and amendments

Representative comments on our draft definition and our responses to them were shown as follows.

1. Organizational interventions

Two experts pointed out that our outcomes were restricted. For example, they advised it would be better to include additional health outcomes or safety. In addition, they suggested “deterioration” was too strong. Furthermore, they commented that organizational interventions may also include actions that indirectly—as well as directly—target working conditions. Finally, in the Notes section, there was a comment that the phrase “Interventions, approaches applied to teams” was incomprehensible.

First, according to their advice, we decided to revise the description of outcomes to be more inclusive. Specifically, we changed “*mental health, physical health, quality of life, and work‐related outcomes of workers*” into “*the highest degree of physical, mental, and social well‐being of workers in all occupations*,” which was based on the definition of occupational health suggested proposed by ILO and WHO.^1^ Second, in light of the point that organizational interventions may include approaches that indirectly target working conditions, we modified the description of organizational interventions, by changing the phrase “planned actions that directly target working conditions” into “planned actions that *primarily* directly target working conditions.” Third, we also revised the phrase “Interventions, approaches applied to teams” into “Interventions may include team‐based approaches” in the Notes section.

2. Participatory elements of organizational interventions

Table 1 presents the summary of the experts' comments. Primarily, the experts gave us the following three suggestions. First, they gave feedback on criterion (1): it seems unrealistic that employees should participate in each step of intervention. For example, the case where employees could join in action planning and implementing, but not in the evaluation process could be excluded from the participatory organizational interventions based on the draft definition we proposed. Second, they offered a comment on criterion (2): it is also incompatible with reality that all the employees in the workplace should participate in the intervention. For example, guided focus groups with selected employees and representatives of management and professionals was a common approach; thus, a more flexible participatory style should be acceptable. Third, they suggested that it may be more suitable to use the term “workers” rather than “employees” because all workers, regardless of the employment relationship, such as day laborers or interns, could participate in an intervention.

**TABLE 1:** Summary of experts' comments on a draft definition of participatory organizational interventions.^a^

Comment 1: It seems rather unrealistic to expect participation of employees in each step, including evaluation. Comment 2: While participation of employees is important, there may be a different intensity of this participation. For example, the use of guided focus groups consisting of selected employees and representatives of management and professionals has been a common approach. Comment 3: From experience, employee participation in the action planning and implementing process could produce positive effects. Comment 4: Involving employees in evaluation seems problematic in terms of independent assessment and related quality standards of intervention research. Comment 5: Flexibility in which parts of the interventions and employees’ participation should be accepted. Comment 6: It also may be essential whether employees participate in the intervention voluntarily or compulsorily, even though it would be difficult to distinguish. Comment 7: The contents of participatory interventions vary among practitioners. Thus, when comparing studies of participatory intervention, we need caution regarding what was done in each intervention. Comment 8: It may be more appropriate to use “workers” than “employees” because all workers, regardless of the employment relationship, such as day laborer, family, intern, or volunteer, should be intervened.

^a^ The authors edited the sentences from experts for clarity.

Based on these comments, we decided to change “employees” into “workers.” In addition, in light of the point that it would be unrealistic that all the workers in the workplace should participate directly in each step of intervention, we thought the case where workers participated in only part of the intervention, or only elected representatives among workers participated in the intervention should also be included in participatory organizational interventions. However, for improving the work environment, providing opportunities for all the workers to participate in organizational interventions is essential.^13^ Also, consultation between the organization and workers should take place at all stages of the intervention.^13^ Then, we thought it would also be necessary that all the workers could have opportunities to participate in every step of the intervention in some form, regardless of direct participation or indirect participation through elected representatives. Accordingly, we revised the draft definition as follows (Table 2).

**TABLE 2:** The final proposed definition of participatory organizational interventions.

1. Organizational interventions *Organizational interventions are planned actions that primarily directly target working conditions with the aim of promoting and maintaining of the highest degree of physical, mental, and social well‐being of workers in all occupations. In addition, organizational interventions are often primary and secondary prevention‐focused, but may also include tertiary prevention*. *Notes* *Interventional actions may target single or multiple known risk factors at work*. *Interventions may include team‐based approaches*. *Interventions may be multimodal, including any combination of individual, manager, and organizational approaches such as workplace mental health promotion with an organizational component*. *Interventions may include tertiary prevention such as helping workers with mental health problems return to work*. *NOT: National, regional or global‐level policies, regulations; interventions which only address individual‐level interventions, or which only address knowledge, awareness, attitudes, stigma reduction for managers, or which only address knowledge, awareness, attitudes, stigma reduction of workers*.
2. Participatory elements of organizational interventions *Workers participate on steps of an intervention, such as action planning, implementing, evaluating, and reviewing the intervention. Ideally, all workers participate in every step of the intervention, while some workers participate in part of the steps of the intervention in some cases*. *In principle, all workers participate in each step of intervention, while it is also acceptable that only elected representatives among workers to participate in the intervention*.

### 3.3 The final proposed definition of participatory organizational interventions

The final proposed definition of organizational interventions and the participatory elements of organizational interventions are as follows.

1. Organizational interventions

*Organizational interventions are planned actions that primarily directly target working conditions with the aim of promoting and maintaining of the highest degree of physical, mental, and social well‐being of workers in all occupations. In addition, organizational interventions are often primary and secondary prevention‐focused, but may also include tertiary prevention*.

*Notes*

- *Interventional actions may target single or multiple known risk factors at work*.
- *Interventions may include team‐based approaches*.
- *Interventions may be multimodal, including any combination of individual, manager, and organizational approaches such as workplace mental health promotion with an organizational component*.
- *Interventions may include tertiary prevention such as helping workers with mental health problems return to work*.
- *NOT: National, regional or global‐level policies, regulations; interventions which only address individual‐level interventions, or which only address knowledge, awareness, attitudes, stigma reduction of managers, or which only address knowledge, awareness, attitudes, stigma reduction of workers*.

2. Participatory elements of organizational interventions

1. *Workers participate on steps of an intervention, such as action planning, implementing, evaluating, and reviewing the intervention. Ideally, all workers participate in every step of the intervention, while participating in part of the steps of the intervention in some cases*.
2. *In principle, all workers participate in each step of intervention, while it is also acceptable that only elected representatives among workers participate in the intervention*.

## 4 DISCUSSION

In this paper, we proposed a definition of participatory organizational interventions based on previous studies and experts' opinions. We believe the definition could be useful for improving safe and healthy working environments. The final proposed definition may facilitate researchers and practitioners conducting participatory organizational interventions in a similar manner while maintaining diversity regarding who is involved and how. Furthermore, it may also be important for workers themselves to practice the interventions on their own initiative based on the definition. The proposed definition may also be useful in conducting systematic reviews and meta‐analyses of participatory organizational interventions in a more comprehensive and integrated way. Such systematic reviews and meta‐analyses would contribute to accumulating evidence for the effectiveness of the intervention.

The proposed definition could be acceptably comprehensive and thus agreeably reflect common views and opinions on organizational interventions and participatory organizational interventions from experts in this field. However, it was interesting that some of these experts expressed views on the heterogeneity of participatory organizational interventions. For example, some argued that workers in principle should participate in every step of the intervention, while others believed that workers may not necessarily participate in all the steps of intervention (Table 1, Comments 1–5). One even suggested that workers should not be involved in the step of reviewing, to ensure independent assessment of the outcomes. Different opinions were also obtained on whether all workers in a target group should be involved in an intervention or whether participation of only part of the workers may be allowed. Additionally, some pointed out that it was important to distinguish between voluntary or forced participation of workers in an intervention, as forced participation may be problematic (Table 1, Comment 6). These comments certainly reflect different views on participatory organizational interventions. While we propose a single common definition, these opinions may provide insight into important dimensions of participatory organizational interventions that can be used to further classify the interventions into subtypes. We encourage researchers to be aware of these characteristics of participatory organizational interventions and describe these in their reports of intervention trials. Comparing implementations and outcomes of participatory organizational interventions with different characteristics may make it possible to identify essential components that may affect the outcome of interventions.

### 4.1 Limitations

This opinion paper has several limitations. First, we collected opinions from the experts only once; in addition, we asked for input from only a limited number of experts. Accordingly, the consensus‐building process should be based on the opinions of a larger number of experts. Second, although we conducted a comprehensive review of relevant literature, the available literature may have been inadequate for developing a draft definition. As a result of publishing this opinion paper, we anticipate further comments and suggestions from various researchers and practitioners for improving our definition.

## AUTHOR CONTRIBUTIONS

Asuka Sakuraya led the drafting of the manuscript. Norito Kawakami, Akizumi Tsutsumi, Kotaro Imamura, and Mako Iida provided critical revision of the manuscript. All authors read and approved the final manuscript.

## DISCLOSURE

*Approval of the research protocol*: N/A; *Informed consent*: N/A; *Registry and the registration no. of the study/trial*: N/A; *Animal studies*: N/A; *Conflict of interest*: The authors ASa, KI, and NK are employed at the Department of Digital Mental Health, an endowment department supported with an unrestricted grant from 15 enterprises (https://dmh.m.u‐tokyo.ac.jp/c), outside the submitted work.

## Data Availability

Data sharing is not applicable to this article as no new data were created or analyzed in this study.

## References

[1] Joint ILO/WHO Committee on Industrial Hygiene Report (1st Meeting, Geneva, 28 August ‐ 2 September 1950) International Labour Office; 1950.

[2] Report of the Joint ILO/WHO Committee on Occupational Health (Twelfth Session, Geneva, 5‐7 April 1995). International Labour Organization; 1995.

[3] Forastieri V. Improving Health in the Workplace: ILO's Framework for Action. International Labour Organization; 2014. Accessed July 3, 2022. https://www.ilo.org/safework/info/publications/WCMS_329350/lang‐‐en/index.htm

[4] C161 Occupational Health Services Convention, 1985. Geneva: International Labour Organization (ILO); 1985.

[5] C155 Occupational Safety and Health Convention, 1981. In Geneva: International Labour Organization (ILO); 1981.

[6] OSH Management System: A tool for continual improvement. International Labour Organization; 2011. 978‐92‐2‐124739‐5 (print), 978‐92‐2‐124740‐1 (web pdf).

[7] Naghieh A , Montgomery P , Bonell CP , Thompson M , Aber JL . Organisational interventions for improving wellbeing and reducing work‐related stress in teachers. Cochrane Database Syst Rev. 2015;(4):Cd010306. doi:10.1002/14651858.CD010306.pub2 25851427 PMC10993096

[8] Joyce K , Pabayo R , Critchley JA , Bambra C , Cochrane Public Health Group . Flexible working conditions and their effects on employee health and wellbeing. Cochrane Database Syst Rev. 2010;2010:Cd008009. 20166100 10.1002/14651858.CD008009.pub2 PMC7175959

[9] Nielsen K , Randall R , Holten A‐L , González ER . Conducting organizational‐level occupational health interventions: what works? Work Stress. 2010;24:234‐259.

[10] Ruotsalainen JH , Verbeek JH , Mariné A , Serra C . Preventing occupational stress in healthcare workers. Cochrane Database Syst Rev. 2015;2015:Cd002892. 25847433 10.1002/14651858.CD002892.pub5 PMC6718215

[11] Tsutsumi A , Shimazu A , Yoshikawa T . Proposed guidelines for primary prevention for mental health at work: an update. Environ Occup Health Pract. 2019;1:2‐12.

[12] Abildgaard JS , Hasson H , von Thiele SU , Løvseth LT , Ala‐Laurinaho A , Nielsen K . Forms of participation: the development and application of a conceptual model of participation in work environment interventions. Econ Ind Democracy. 2018;41:746‐769.

[13] ISO 45003:2021(en) Occupational health and safety management . Psychological Health and Safety at Work. Guidelines for Managing Psychosocial Risks. International Organization for Standardization; 2021. Accessed July 3, 2022. https://www.iso.org/obp/ui/#iso:std:iso:45003:ed‐1:v1:en

[14] Llorens‐Serrano C , Pérez‐Franco J , Oudyk J , et al. COPSOQ III. Guidelines and questionnaire; 2019. Accessed November 16, 2021. https://www.copsoq‐network.org/guidelinesandquestionnaire/

[15] Dahl‐Jørgensen C , Saksvik PO . The impact of two organizational interventions on the health of service sector workers. Int J Health Serv. 2005;35:529‐549. 16119574 10.2190/P67F-3U5Y-3DDW-MGT1

[16] Tsutsumi A , Nagami M , Yoshikawa T , Kogi K , Kawakami N . Participatory intervention for workplace improvements on mental health and job performance among blue‐collar workers: a cluster randomized controlled trial. J Occup Environ Med. 2009;51:554‐563. 19365287 10.1097/JOM.0b013e3181a24d28

[17] Uchiyama A , Odagiri Y , Ohya Y , Takamiya T , Inoue S , Shimomitsu T . Effect on mental health of a participatory intervention to improve psychosocial work environment: a cluster randomized controlled trial among nurses. J Occup Health. 2013;55:173‐183. 23585499 10.1539/joh.12-0228-oa

[18] WHO guidelines on mental health at work . World Health Organization; 2022. 36395303

